# The Quality of Carcass and Meat from Geese Fed Diets with or without Soybean Meal

**DOI:** 10.3390/ani10020200

**Published:** 2020-01-25

**Authors:** Jakub Biesek, Joanna Kuźniacka, Mirosław Banaszak, Marek Adamski

**Affiliations:** Department of Animal Breeding, Faculty of Animal Breeding and Biology, UTP—University of Science and Technology in Bydgoszcz, Mazowiecka 28, 85-084 Bydgoszcz, Poland; kuzniacka@utp.edu.pl (J.K.); miroslaw.banaszak@utp.edu.pl (M.B.); adamski@utp.edu.pl (M.A.)

**Keywords:** drip loss, goose, growth, meat quality, muscles, physicochemical traits, protein, yellow lupin

## Abstract

**Simple Summary:**

This research explains how yellow lupin, potato protein, and brewer’s yeast in diets affect goose carcass and meat quality. The study showed no negative effect of yellow lupin on most traits, excluding leg muscle weight and content in carcass. Meat from geese fed with yellow lupin was characterized by better suitability for further technological processing, which was confirmed by the analysis of the ability to keep water in meat (drip loss). The results obtained show that the use of yellow lupin in diets for geese can be an alternative to soybean meal. Soybean is mainly a genetically modified material. Consumers expect non-genetically modified products. Yellow lupin as a protein source in geese diets gives wider possibilities and choices for the market, and it can support further studies. It has been shown that the use of yellow lupin in geese diets allows fattening by oats to continue, especially in small-scale family farms where feed produced from their own agricultural crops is often used.

**Abstract:**

The aim of the study was to compare the carcass and meat quality of geese fed with soybean meal or yellow lupin. In total, 210 White Kołuda^®^ geese were divided into 2 groups (1, soybean meal (SBM); 2, yellow lupin (YL), potato protein, and brewer’s yeast) of 5 replications (21 birds per each). After 16 weeks, 10 geese (5 females, 5 males) from each group were slaughtered. Carcass dissection was done, and major physicochemical traits were analysed (pH, water holding capacity, drip loss, color, and chemical composition of muscles). Weight of leg muscles and their proportion in the carcass were higher (*p* < 0.05) in SBM. Breast muscles from SBM were characterized by increased (*p* < 0.05) drip loss, enhanced (*p* < 0.05) content of protein, water, collagen and connective tissue, and lower (*p* < 0.05) fat content. Leg muscles from SBM were characterized by higher (*p* < 0.05) protein and water content but decreased (*p* < 0.05) salt and fat content compared to YL. The addition of YL (approx. 28%), potato protein, and brewer’s yeast had no negative effect on most meat traits and could partly replace SBM as a protein source in geese feeding. Hence, yellow lupin, potato protein and brewer’s yeast can be used in geese rearing followed by fattening with oats. Some producers do not have the option of using soybean meal. Small-scale farms use their own crop resources, so lupins can be an alternative source of protein for soybean meal.

## 1. Introduction

Carcass and meat quality in broiler poultry depends on many parameters, including genotype, sex, age, and diet. In the composition of feeds, the content of total protein affects the nutritive value of muscle tissue [1]. Most broiler geese produced in Poland are two-strain crossbreeds designated W31 (White Kołuda^®^ geese) and they make up over 95% of the local population. These birds are reared for 13 weeks with a normal feed mixture and for the next 3 weeks before slaughter (week 16), according to the traditional method of production technology, they are fattened only on oat grains [2,3]. The basic source of protein in poultry feeds is imported soybean meal (mainly GMO). Its high-protein component is unrivalled as it contains 45% to 55% crude protein [4]. Because of the need to secure the supply of protein to animals and people, as well as the existing ban on the distribution of feed from genetically modified plants in Poland, the use of legumes as an alternative to soybean has been attracting growing interest [5,6]. Among alternative plants are lupin species that contain 35–40% of protein and 8–12% fat. However, lupin plants are poor in digestible carbohydrates, i.e., oligosaccharides and starch. In the past, the use of lupins in livestock diets was limited by high levels of alkaloids, tannins, and non-starch polysaccharides (NSPs), which reduced palatability, nutritional value, and feed intake [7,8,9,10]. New varieties of lupin, however, created as a result of breeding studies, are characterized by a lower content of anti-nutritional ingredients and no longer have such drawbacks [11,12,13,14,15].

The aim of this study was to compare the productivity parameters and quality of carcass and meat from geese receiving balanced feed containing protein sourced from yellow lupin (at 28% content in feed), potato protein, and brewer’s yeast, as an alternative to soybean meal.

The tested hypothesis is: Yellow lupin seeds, potato protein, and brewer’s yeast used as a high-protein component as an alternative for soybean meal in complete feed positively influences the quality of meat from broiler geese during the 13 weeks before traditional oat fattening can be started. This study also indicates the possibility of economically positive rearing using yellow lupin seed at a 28% level in feed and then continuing with traditional oat fattening.

## 2. Materials and Methods

According to Polish law and the EU directive (no. 2010/63/EU), the experiment did not require approval from the Local Ethical Committee as it was done by local farmers on a small scale (in the production conditions). The main part of the experiment started after the slaughter when the raw material was provided. This was an implementation experiment (practical tests, after the experimental stage).

### 2.1. Animals and Diets

The study was conducted with 210 White Kołuda^®^ geese, divided into 2 groups, with 105 birds in each group and 5 replications (21 birds each). Group 1 (control) received balanced feed containing soybean meal, while Group 2 (experimental) received balanced feed containing yellow lupin cv. Mister (ground form), potato protein, and brewer’s yeast (without soybean meal). The birds were not assigned to groups based on male and female sex because differences due to sexual dimorphism are not noticed in the rearing period of broiler geese. Female and male White Kołuda^®^ geese are two-breed hybrids, constituting slaughter material, and are characterized by uniform growth and non-differing slaughter performance. Each goose was marked with a padlock mark and weighed individually. In the experiment, two geese sexes were used because it was during a normal production cycle on a small-scale farm, where producers aim to achieve a good quality product regardless of the sex of the birds. The diet of the birds differed in the three periods of rearing, where the concentrate level decreased from 50% in the first stage to 40% in the second stage. Herein, the content of wheat in the feed increased from 50% to 60%. Hence, there was 20.51%–20.52% of crude protein (CP) and 11.54–11.55 MJ of metabolic energy (ME) per kg of feed in the first period; the second period comprised 18.01–18.03% of CP and 11.65–11.67 MJ/kg of feed (Table 1). The composition of concentrates and its analysed nutritive value are presented in Table 2. Information on the concentrates was provided by the animal feed manufacturer.

The birds were reared for 16 weeks, and in the last 3 weeks before slaughter, they were fattened on oat grains. The feed was given ad libitum for the entire goose maintenance period. Fattening on oats is the traditional method of geese production in Poland. Oat fattening causes an increase in fat, which is designed to give the goose meat a typical taste and a unique structure. Each goose ate approximately 6.5 kg of oats for the fattening period. Up to the age of 6 weeks, the geese were kept in pens indoors, and then they moved to free range. The pens were prepared in accordance with applicable standards, where the density was 19 kg of geese per 1 m^2^. After 6 weeks, when the birds developed thermoregulation capacity and completed the process of buyer gland development, they were transferred in accordance with the established groups and replications to the free range, where the maximum density was up to 6.5 kg of goose per 1 m^2^. This is also associated with a feather cover that closes in geese after 6 weeks of age. Geese could be moved to free range at an earlier date, however weather conditions could result in rearing failure. For the first 4 weeks, an artificial heating system was used in the building, where the temperature was 32 degrees Celsius and gradually decreased to 22. For the next 2 weeks, the temperature in the building fluctuated between 22 and 24 degrees Celsius. After 6 weeks, the environmental conditions were natural (free range) and they fluctuated (summer months). The lighting program was in line with goose production technology. For the first 3 days geese had constant lighting, while lighting was applied for 16 h the remaining 5.5 weeks. After 6 weeks, the lighting was natural, which, in the summer, was about 16 to 12 h (period from June to September).

### 2.2. Productivity Parameters

Birds and feed refusals were weighed. Birds were weighed individually. Each bird had a padlock badge, thanks to which body weight was controlled for each individual, and the average result for the whole group was accurately calculated. On this basis, the mean values of 1-day-old chicks and the final body weight (BW), body weight gain (BWG), total feed intake (FI), and feed conversion ratio (FCR) per kg of body weight gain for the whole herd were calculated.

### 2.3. Meat Traits

After 16 weeks of rearing, 20 birds (10 from each group: 5 males and 5 females), randomly selected, were slaughtered. Each bird was taken as a unit in every group comprising the obtained experimental results. Therefore, the mean values were represented by 5 replications with 2 geese each (10 birds). The plucked and gutted carcasses were analyzed for qualitative parameters. The pH value of breast muscles was first measured 15 min post-mortem (pH_15_). The carcasses were then placed in cold storage at 2 °C, and pH was measured again after 24 h (pH_24_) utilizing a CX-701 pH-meter with a knife electrode (Elmetron, Zabrze, Poland). The carcasses were weighed on Radwag scales (Radwag, Radom, Poland) with accuracy to the nearest 0.01 g. Next, the carcasses were dissected by applying the method described by Ziołecki and Doruchowski [16], and the following parts were separated: breast muscles, leg muscles, skin with subcutaneous fat, abdominal fat, offal (liver, heart, stomach), wings with skin, neck with skin (cut off between the last cervical vertebra and the first thoracic vertebra of spine), and carcass remains (trunk, leg bones). Each carcass part was weighed, and dressing percentage was calculated by the formula WC/BW × 100%, where WC is the weight of the carcass and BW is body weight. The color of breast and leg muscles was assessed using a Konica Minolta colorimeter (CR400, Tokyo, Japan), calibrated using the white calibration plate no. 21033065 and the D_65_ Y_86 1_ x_0 3188_ y_0 3362_ scale. The color was graded according to the CIELab (Comission Internationale de l’Eclairage—International Commission of Lighting) system for L* (lightness), a* (redness), and b* (yellowness) [17]. Breast muscles were also tested for drip loss. For that purpose, breast muscles were weighed post-mortem (M1) and after 24 h of storage in a cold room at 2 °C (M2) [18]. Calculations were done using the formula DP = 100% − (M2/M1) × 100%. Breast and leg muscles were also analyzed for (WHC) water holding capacity [19]. Herein, muscles were homogenized in a mincer (homogenizer). Pooled samples of about 0.300 g with 5% standard deviation, weighted on an accurate analytical balance (M1) were wrapped in Whatman grade 1 filter paper and kept under 2 kg pressure for 5 min (M2). The water holding capacity of meat was calculated based on the difference in weight before and after the test (WHC = 100% − (M2/M1) × 100%).

### 2.4. Chemical Composition of Meat

Pooled samples of homogenized breast and leg muscles (90 g) from each study group were analyzed in a laboratory for the content of protein, collagen, salt, connective tissue, fat and water according to the PN-A-82109:2010 standard, using a FoodScan apparatus (FOSS, Warsaw, Poland) with a Near Infrared Transmission (NIT) spectrometer calibrated for an artificial neural network (ANN).

### 2.5. Statistical Analysis

Numerical data were analyzed via statistical software [20] by calculating the mean values (x) of the examined parameters and their standard deviations (SD) with the dietary high-protein compound as the main factor (with or without soybean meal). Standard error of the mean (SEM) was also calculated. The significance of differences was verified by a Student’s *t*-test at the significance level of *p*-value < 0.05. Differences between groups were statistically significant when the *p*-value was less than 0.05. Each bird was the basic unit in every group comprising the obtained experimental results of meat quality (each group: 10 birds = 2/pen). The productivity parameters were calculated for the whole herd of geese (each group: 105 birds).

## 3. Results

Analysis of BW showed no significantly higher (*p* > 0.05) values in Group 1 (soybean meal) compared to Group 2 (without soybean meal). The average BWG of geese from the 1st group was 0.52 g higher than that in the 2nd group. Considering the whole rearing period, FI in Group 1 was 0.09 kg lower than that in Group 2. FCR per kg of weight gain in birds from Group 1 was 0.24 kg lower than that in Group 2. No statistically significant differences (*p* > 0.05) of productivity parameters between groups were found (Table 3).

The analysis of post-mortem parameters (Table 4) demonstrated that the pre-slaughter BW, the weight of the gutted carcass, and dressing percentage in both groups were similar (*p* > 0.05), where the mean values for both groups were 6483.5 g, 4355.2 g, and 67.2%, respectively. There were also no significant differences (*p* > 0.05) between both study groups with respect to the other characteristics, such as the weight of the neck, wings, offal, or carcass remains. Table 5 shows that the weight of leg muscles and their proportion in the carcass significantly increased (*p* = 0.043, *p* = 0.036, respectively) in geese from Group 1 compared to that of Group 2. The values of other parameters of muscles and carcass fatness were similar (*p* > 0.05) in both groups. Physicochemical analysis of breast muscles from geese did not reveal any significant differences (*p* > 0.05) in pH, WHC or parameters of muscle color (Table 6). However, the drip loss from breast muscles significantly increased (*p* = 0.007) in birds from Group 1.

Breast muscles from Group 1 also contained higher amounts of protein (*p* < 0.001), water (*p* < 0.001), collagen (*p* = 0.013), and connective tissue (*p* = 0.026), but less intramuscular fat (*p* = 0.000), than those from Group 2. Values of WHC and color parameters (L*, a*, b*) for leg muscles were similar (*p* > 0.05) in both study groups (Table 7). However, leg muscles from geese in Group 1 contained significantly higher amounts of protein (*p* < 0.001) and water (*p* < 0.001) compared to that of Group 2. Moreover, the content of salt (*p* < 0.001) and intramuscular fat (*p* < 0.001) in leg muscles from Group 2 was higher (0.94% and 8.93%), than that in Group 1 (0.81% and 8.21%).

## 4. Discussion

The use of lupin species in geese diets and its impact on productivity parameters and quality of meat have been investigated by other researchers, including Bieliński et al. [21], Biesiada-Drzazga et al. [22], Biesiada-Drzazga [23], and Pietrzak et al. [24]. Scientific research previously carried out in the field on the use of different sources of protein in goose nutrition was conducted in the form of scientific experiments, where rearing was not commercial and ended before the beginning of the oat grain fattening period. Research on goose meat quality is limited, and literature is hardly available. By conducting our research, we wanted to show the results regarding the quality of the finished raw material, produced based on traditional fattening by oat grains (good quality meat and fat with excellent sensory properties [2]).

In our work, feed rations were done according to recommendations by Smulikowska and Rutkowski [25], that is, 15.0–22.0% of crude protein and 11.7–12.0 MJ of metabolic energy per kg of feed. Bieliński et al. [21] added sweet lupin to the diet of Italian white geese at a level of 12–15% of daily rations and reported, as in our study, no negative effects on the productivity parameters of geese during the 10-week rearing period. They also found that this proportion of lupin in feed had no negative effect on weight gain or egg production in geese. Similar conclusions were reached by Biesiada-Drzazga et al. [22], who replaced soybean meal with yellow lupin at a level of 25–50% of the daily ration. In their experimental study, geese were reared for 10 weeks, and there was no deterioration in BWG, weight of carcass, breast muscles, or leg muscles in geese on a diet without soybean meal compared to the control birds. In another experiment [23], geese that received feed in which soybean meal was partly replaced with yellow lupin and sunflower seed meal were characterized by significantly lower fat content in the carcass and lower weight of breast muscle compared to the control group. Slightly different results were obtained in our research, where the weight of leg muscles and their proportion in the carcass was lower in geese fed with yellow lupin in comparison to the group fed with soybean meal. The effect of diets containing yellow lupin (10% per ration) on the quality of the carcass in White Kołuda^®^ geese was also investigated by Pietrzak et al. [24]. The authors, similarly to our study, found no negative effect of yellow lupin on most of the carcass parameters, and the values of meat traits in geese were like those reported from our experiments. Muscle results are not necessarily caused by a change of feed and by other protein components, because the protein content (amount), and hence muscle growth, is genetically determined. However, the fat content of the carcass and muscle (intramuscular fat) may change due to nutrition.

The results of our study show that the addition of yellow lupin to goose feeds did not affect the color of breast and leg muscles (L*, a*, b*), but it caused differences in the chemical composition of meat between the analyzed groups. The mean values of the basic chemical components of breast and leg muscles, lightness (L*), and yellowness (b*) of breast and leg muscles were similar to the values reported by Pietrzak et al. [24]. However, the redness of meat (a*) measured in our study (10.96 to 13.71) was much lower than the values reported by Pietrzak et al. [24] (22.30 to 23.45). This could indicate that birds had various contents of red muscle fibres. The higher values of yellowness could indicate higher levels of intramuscular fat. The differences between various meat color studies may relate to the changes of pH values and the glycolysis process [26]. The differences in color may also be affected by the structure of muscle fibers or the method of calibration and the type of colorimeter used to perform the analysis [24]. Many factors influence meat color results. Muscle tissue has a dye—myoglobin. Its content depends on many factors such as the species, age, or condition of the animal. In our research, the geese came from one hatching and were also characterized by a similar body condition. Another factor may be the post-slaughter period, during which post-mortem concentration and pH decrease (acidification increases). A pH value that decreases indicates the proper course of glycolysis in muscle tissue [27]. Higher pH of meat is often associated with a higher water-holding capacity. According to Zhuang and Savage [28] meat with a higher L* value and lower pH value is characterized by lighter color, and this was confirmed in our own study. In turn, pH below 5.7 is characterized by poorer meat suitability for further technological processing. This could be due to a PSE (pale, soft, exudative) defect. However, waterfowl, including geese, are characterized by a large proportion of red muscle fibers, which means that PSE is very rare. A higher drip loss in breast muscles in the control group in the present study may be associated with a higher content of protein in the meat [24].

Drip loss was different in the analysed groups and was lowest for breast muscles from geese receiving feed in which protein was balanced without soybean meal. According to Augustyńska-Prejsnar and Sokołowicz [29], lower values of drip loss indicate better juiciness of muscles. This trait, associated with the capacity of muscles to hold water, has a huge impact on meat quality and depends on the genotype, post-mortem chemical changes in meat, and the way meat is stored [30,31]. Pietrzak et al. [24] suggested that differences in the values of drip loss may be caused by different contents of protein in muscles. Consumers require juicy meat, which is why the ability to keep water is such an important element of quality. After slaughter, the meat is prepared and distributed to stores. Meat should, despite a given shelf life in stores, be characterized by high firmness and low drip loss. Our study revealed a higher proportion of fat in leg and breast muscles from Group 2 reared on a diet without soybean meal. According to Tyra and Mitka [32], this may indicate better palatability of meat because fat is the carrier of flavours. Geese (especially two-breed hybrids like the White Kołuda^®^ goose) are also characterized by a favorable fatty acid profile in fat, which is why the increased fat content in carcass and muscles can be considered a desirable trait. In addition, the traditional technology of fattening geese on oats has, among other factors, contributed to increasing fatness in geese carcasses. Other researchers investigating this problem [25,33] proposed a much lower addition of yellow lupin (5% to 15%) as optimal without deteriorating productivity parameters. Literature on the quality of goose meat is quite limited in terms of the use of various sources of protein in feed. Many scientists have been involved in comparing goose meat quality according to strains and housing systems [34,35,36].

## 5. Conclusions

The addition of yellow lupin, potato protein, and brewer’s yeast to goose feed used in our experiment (about 28%) had no negative effects on most meat traits or physicochemical characteristics of muscles from these birds, excluding leg muscle weight and content in carcass. The ability to retain water in goose meat showed better suitability for further technological processing. Yellow lupin can be proposed as an alternative to soybean meal. The use of yellow lupin seeds, potato protein, and brewer’s yeast in geese diets could be included in production practices, especially in small-scale farms with semi-intensive systems of rearing. This diet allows geese to be reared and fed properly before the traditional method of fattening by oats is employed. Use of alternative protein sources could determine wider and different types of products for producers and for the consumer market.

## Figures and Tables

**Table 1:** Proportion of concentrate and wheat in feed for geese.

**Weeks 1 to 6 of Rearing**	**Concentrate**	**Wheat**
Control group (1)	50%	50%
Experimental group (2)	50%	50%
**Weeks 7 to 13 of rearing**	**Concentrate**	**Wheat**
Control group (1)	40%	60%
Experimental group (2)	40%	60%
**Weeks 1 to 6 of rearing ^1^**		
CP (%) ^2^	20.51–20.52	
ME (MJ/kg of feed) ^3^	11.54–11.55	
**Weeks 7 to 13 of rearing**		
CP (%)	18.01–18.03	
ME (MJ/kg of feed)	11.65–11.67	
**Weeks 13 to 16 of rearing (1, 2 groups)**	**fattening on oats (*ad libitum*)**	
CP (%)	8.90	
ME (MJ/kg of feed)	10.20	

^1^ values of crude protein and metabolic energy that were declared from the producer; ^2^ crude protein (%); ^3^ metabolic energy (MJ/kg of feed).

**Table 2:** Composition of concentrate and its analysed nutritive value for geese.

Composition, %	Group	
	1 ^1^	2 ^2^
Soybean meal, 44%	65	-
Yellow lupin cv. Mister, 42.5%	-	68.98
Potato protein	-	3
Brewer’s yeast	-	3
Triticale	23.04	12
Soybean oil	5.2	5.4
Premix 1%	2	2
Chalk fodder, CaCO_3_	2	2
CaH_4_P_2_O_8_	1.52	1.74
NaHCO^3^	0.84	0.8
Fodder salt, NaCl	0.18	0.12
L-lysine	-	0.32
DL-methionine	0.2	0.4
L-threonine	0.02	0.24
Crude protein	31.42	31.43
Metabolic energy, MJ/kg	10.79	10.79
Calcium, %	1.92	1.92
P-available, %	0.56	0.56
Lysine, %	1.82	1.82
Threonine, %	1.28	1.28
Valine, %	1.14	1.14

^1^ control group fed with soybean meal; ^2^ experimental group fed with yellow lupin seeds, potato protein, and brewer’s yeast.

**Table 3:** Productivity parameters of all of geese (x; ±SD; SEM) after the 16-week rearing period.

Traits	Group 1 ^1^	Group 2 ^2^	SEM	*p*-Value
*n* = 105 per Group	X ± SD	X ± SD		
Body weight of 1-day-old chicks (g)	100.05 ± 9.77	99.21 ± 7.91	0.42	0.621
Final body weight (g)	6205.90 ± 66.00	6144.86 ± 88.10	30.52	0.574
Body weight gain (g/day)	53.09 ± 5.76	52.57 ± 7.65	0.260	0.580
Total feed intake (kg)	27.38 ± 0.62	27.62 ± 0.60	0.19	0.565
Feed conversion ratio (kg/kg gain)	4.41 ± 0.10	4.50 ± 0.10	0.53	0.567

*n*, means represented by 105 birds (21 birds chosen from 5 replications), each bird was a basic experimental unit for means results; ^1^ control group, fed with feed based on soybean meal; ^2^ experimental group, fed with yellow lupin seeds, potato protein, and brewer’s yeast; x, means represented by 5 replications with 21 geese per each (105 birds); ±SD, standard deviation; SEM, standard error of the mean. Each bird was the basic unit in every group comprising the obtained experimental results.

**Table 4:** Post-mortem parameters (mean; ±SD; SEM) in 16-week-old geese.

Traits		Group 1 ^1^	Group 2 ^2^	SEM	*p*-Value
*n* = 10 per Group		X ± SD	X ± SD		
Pre-slaughter body weight (g)		6519.00 ± 109.79	6448.00 ± 148.38	29.55	0.240
Weight of carcass (g)		4340.69 ± 162.45	4369.66 ± 136.30	32.80	0.671
Dressing percentage (%)		66.58 ± 2.15	67.78 ± 2.14	0.49	0.226
Weight and proportion in carcass					
	Neck with skin (g)	386.18 ± 73.96	355.83 ± 48.61	14.06	0.292
	Neck with skin (%)	8.91 ± 1.76	8.15 ± 1.10	0.33	0.258
	Wings (g)	570.16 ± 55.54	545.61 ± 47.54	11.60	0.302
	Wings (%)	13.16 ± 1.51	12.51 ± 1.27	0.31	0.308
Weight of offal (g)		357.29 ± 33.65	347.54 ± 25.22	6.57	0.473
Weight of remains (g)		997.00 ± 200.80	1130.99 ± 158.55	42.27	0.115

*n*, means represented by 10 birds (2 birds chosen from 5 replications), each bird was a basic experimental unit for means results; ^1^ control group, fed with feed based on soybean meal; ^2^ experimental group, fed with yellow lupin seeds, potato protein, and brewer’s yeast; x, means represented by 5 replications with 2 geese per each (10 birds); ±SD, standard deviation; SEM, standard error of the mean.

**Table 5:** Muscles and fat (mean; ±SD; SEM) in 16-week-old geese.

Traits		Group 1 ^1^	Group 2 ^2^	SEM	*p*-Value
*n* = 10 per Group		X ± SD	X ± SD		
Weight and proportion in carcass	Breast muscles (g)	632.93 ± 69.80	630.25 ± 60.96	14.27	0.928
	Breast muscles (%)	14.59 ± 1.61	14.42 ± 1.38	0.33	0.811
	Leg muscles (g)	518.54 ± 41.20	470.63 ± 46.93	12.02	0.043
	Leg muscles (%)	12.00 ± 1.26	10.78 ± 1.07	0.29	0.036
	Total muscles (g) ^3^	1151.47 ± 97.45	1100.88 ± 95.34	21.77	0.256
	Total muscles (%)	26.55 ± 2.36	25.20 ± 2.16	0.52	0.200
	Skin with subcutaneous fat (g)	1170.74 ± 138.80	1143.03 ± 109.51	27.39	0.626
	Skin with subcutaneous fat (%)	26.98 ± 3.19	26.15 ± 2.31	0.61	0.517
	Abdominal fat (g)	205.55 ± 51.51	223.88 ± 47.41	10.98	0.418
	Abdominal fat (%)	4.73 ± 1.17	5.13 ± 1.10	0.25	0.450

Statistically significant differences found at the level *p*-value < 0.05; *n*, means represented by 10 birds (2 birds chosen from 5 replications), each bird was a basic experimental unit for means results; ^1^ control group, fed with feed based on soybean meal; ^2^ experimental group, fed with yellow lupin seeds, potato protein, and brewer’s yeast; ^3^ total muscles = breast muscles + leg muscles; x, means represented by 5 replications with 2 geese per each (10 birds); ±SD, standard deviation; SEM, standard error of the mean.

**Table 6:** Physicochemical parameters of breast muscles (means; ±SD; SEM) in 16-week-old geese.

Traits	Group 1 ^1^	Group 2 ^2^	SEM	*p*-Value
*n* = 10 per Group	x ± SD	x ± SD		
pH_15_	6.30 ± 0.19	6.36 ± 0.13	0.04	0.412
pH_24_	6.22 ± 0.45	6.24 ± 0.18	0.08	0.354
Color ^3^				
L*	41.92 ± 3.95	41.06 ± 2.54	0.73	0.571
a*	13.68 ± 1.30	13.71 ± 1.12	0.26	0.961
b*	4.70 ± 1.86	3.92 ± 1.73	0.55	0.346
WHC (%) ^4^	26.20 ± 4.37	29.75 ± 4.71	1.07	0.098
Drip loss (%)	0.63 ± 0.22	0.33 ± 0.21	0.06	0.007
Protein (%)	22.13 ± 0.05	21.77 ± 0.04	0.04	<0.001
Collagen (%)	1.34 ± 0.09	1.19 ± 0.14	0.03	0.013
Salt (%)	1.08 ± 0.05	1.08 ± 0.08	0.01	0.949
Connective tissue (%)	6.04 ± 0.42	5.46 ± 0.64	0.14	0.026
Intramuscular fat (%)	3.09 ± 0.04	3.91 ± 0.02	0.09	<0.001
Water (%)	73.70 ± 0.04	72.48 ± 0.05	0.14	<0.001

Statistically significant differences found at the level *p*-value < 0.05; *n*, means represented by 10 birds (2 birds chosen from 5 replications), each bird was a basic experimental unit for means results; ^1^ control group, fed with feed based on soybean meal; ^2^ experimental group, fed with yellow lupin, potato protein, and brewer’s yeast; ^3^ L*, lightness, a*, redness, b*, yellowness; ^4^ WHC, water holding capacity; x, means represented by 5 replications with 2 geese per each (10 birds); ±SD, standard deviation; SEM, standard error of the mean.

**Table 7:** Physicochemical parameters of leg muscles (mean; ±SD; SEM) in 16-week-old geese.

Traits	Group 1 ^1^	Group 2 ^2^	SEM	*p*-Value
*n* = 10 per Group	x ± SD	x ± SD		
Color ^3^				
L*	39.49 ± 1.14	39.31 ± 3.86	0.62	0.890
a*	11.86 ± 2.62	10.96 ± 2.69	0.59	0.461
b*	2.93 ± 1.64	1.88 ± 1.80	0.39	0.190
WHC (%) ^4^	30.31 ± 0.81	32.03 ± 3.80	0.63	0.180
Protein (%)	19.05 ± 0.05	18.89 ± 0.03	0.02	<0.001
Collagen (%)	1.39 ± 0.13	1.46 ± 0.10	0.03	0.155
Salt (%)	0.81 ± 0.04	0.94 ± 0.03	0.01	<0.001
Connective tissue (%)	7.27 ± 0.66	7.75 ± 0.55	0.14	0.098
Intramuscular fat (%)	8.21 ± 0.01	8.93 ± 0.05	0.08	<0.001
Water (%)	71.15 ± 0.09	70.13 ± 0.09	0.12	<0.001

Statistically significant differences found at the level *p*-value < 0.05; *n*—means represented by 10 birds (2 birds chosen from 5 replications), each bird was a basic experimental unit for means results; ^1^ control group, fed with feed based on soybean meal; ^2^ experimental group, fed with feed without soybean meal; ^3^ L*, lightness, a*, redness, b*, yellowness; ^4^ WHC, water holding capacity; x, means represented by 5 replications with 2 geese per each (10 birds); ±SD, standard deviation; SEM, standard error of the mean;

## References

[1] Kuźniacka J., Adamski M., Czarnecki R., Banaszak M. (2014). Results of Rearing Broiler Chickens Under Various Systems. J. Agric. Sci..

[2] Buzała M., Adamski M., Janicki B. (2014). Characteristic of performance traits and the quality of meat and fat in Polish oat geese. World Poult. Sci. J..

[3] Lewko L., Gornowicz E., Pietrzak M., Korol W. (2017). The effect of origin, sex and feeding on sensory evaluation and some quality characteristics of goose meat from Polish native flocks. Ann. Anim. Sci..

[4] Kaczmarek S.A., Hejdysz M., Kubiś M., Rutkowski A. (2016). Influence of graded inclusion of white lupin (*Lupinus albus*) meal on performance, nutrient digestibility and intestinal morphology of broiler chickens. Br. Poult. Sci..

[5] Księżak J., Święcicki W., Szukała J., Rutkowski A., Jerzak M., Barszczewski J. (2015). Ulepszanie Krajowych Źródeł Białka Roślinnego, Ich Produkcji, Systemu Obrotu i Wykorzystania w Paszach. Raport Końcowy z Realizacji Programu Wieloletniego 2011–2015.

[6] Sońta M., Rekiel A. (2017). Production and use of legumes for feed purposes. Vol. II. The use of fabaceae in animal nutrition. Breed. Rev..

[7] Jezierny D., Mosenthin R., Bauer E. (2010). The use of grain legumes as a protein source in pig nutrition: A review. Anim. Feed Sci. Technol..

[8] Chiofalo B., Lo Presti V., Chiofalo V., Gresta F. (2012). The productive traits, fatty acid profile and nutritional indices of three lupin (*Lupinus* spp.) species cultivated in a Mediterranean environment for the livestock. Anim. Feed Sci. Technol..

[9] Chilomer K., Kasprowicz-Potocka M., Gulewicz P., Frankiewicz A. (2013). The influence of lupin seed germination on the chemical composition and standardized ileal digestibility of protein and amino acids in pigs. J. Anim. Physiol. Anim. Nutr..

[10] Bähr M., Fechner A., Hasenkopf K., Mittermaier S., Jahreis G. (2014). Chemical composition of dehulled seeds of selected lupin cultivars in comparison to pea and soya bean. LWT Food Sci. Technol..

[11] Milczarek A., Osek M. (2019). Effectiveness evaluation of use of various protein feeds for broiler chicken feeding. Ann. Anim. Sci..

[12] Jansen G., Jürgens H.U., Schliephake E., Seddig S., Ordon F. (2015). Effects of growing system and season on the alkaloid content and yield of different sweet L. angustifolius genotypes. J. Appl. Bot. Food Qual..

[13] Hejdysz M., Kaczmarek S.A., Rutkowski A. (2016). Extrusion cooking improves the metabolizable energy of faba beans and the amino acid digestibility in broilers. Anim. Feed Sci. Technol..

[14] Rutkowski A., Kaczmarek S.A., Hejdysz M., Jamroz D. (2016). Effect of extrusion on nutrients digestibility, metabolizable energy and nutritional value of yellow lupine seeds for broiler chickens. Ann. Anim. Sci..

[15] Kaczmarek S.A., Hejdysz M., Kubiś M., Kasprowicz-Potocka M., Rutkowski M. (2016). The nutritional value of yellow lupin (*Lupinus luteus* L.) for broilers. Anim. Feed Sci. Technol..

[16] Ziołecki J., Doruchowski W. (1989). Methods for Assessing Slaughter Value.

[17] CIE (1986). Colorimetry.

[18] Honikel K.O. (1987). The water binding of meat. Fleischwirtschaft.

[19] Grau R., Hamm R. (1952). Eine einfache Methode zur Bestimmung der Wasserbindung in Fleisch. Fleischwirtschaft.

[20] STATISTICA PL (2011). Version 10.0, Series 1101.

[21] Bieliński K., Skarżyński Ł., Pakulska E. (1982). Seeds of faba bean, peas and sweet lupins, as well as flax and rapeseed meal as a source of protein in goose nutrition. Rocz. Nauk. Zootech..

[22] Biesiada-Drzazga B., Górski J., Górska A. (2006). Analysis of slaughter value and muscle fibre thickness of selected muscles in geese broilers as related to feeding applied during the rearing period. Anim. Sci. Pap. Rep..

[23] Biesiada-Drzazga B. (2008). Impact of goose feeding on mixtures containing post-extraction sunflower meal and yellow lupine meal on the quality of muscle and fat tissue. Rocz. Inst. Przem. Mięsn. Tłuszcz..

[24] Pietrzak D., Mierzejewska E., Mroczek J., Michalczuk M., Damaziak K., Makarski M., Adamczak L. (2013). Impact of nutrition and sex on selected quality characteristics of White Koluda Geese meat. Zesz. Probl. Postępów Nauk Rol..

[25] Smulikowska S., Rutkowski A. (2018). Recommended Allowances and Nutritive Value of Feedstuffs. Poultry Feeding Standards.

[26] Dransfilied E., Sosnicki A.A. (1999). Relationship between Muscle Growth and Poultry Meat Quality. Poult. Sci..

[27] Honikiel K.O. (1998). Reference methods for the assessment of physical characteristics of meat. Meat Sci..

[28] Zhung H., Savage E.M. (2010). Comparisons of sensory descriptive flavor and texture profiles of cooked broiler breast fillets categorized by raw meat color lightness values. Poult. Sci..

[29] Augustyńska-Prejsnar A., Sokołowicz Z. (2018). The influence of breed and heat treatment on the quality of pectoral muscles of hens from organic farming after the first year of laying use. Zywn. Nauka Technol. Jakość.

[30] Cheng Q., Sun D.W. (2008). Factors Affecting the Water Holding Capacity of Red Meat Products: A Review of Recent Research Advances. Crit. Rev. Food Sci. Nutr..

[31] Damaziak K., Stelmasiak A., Michalczuk M., Wyrwisz J., Moczkowska M., Marcinkowska-Lesiak M.M., Niemiec J., Wierzbicka A. (2016). Analysis of storage possibility of raw and smoked breast meat of oat-fattened White Kołuda^®^ goose based on their quality characteristics. Poult. Sci..

[32] Tyra M., Mitka I. (2015). The role of intramuscular fat (IMF) in shaping meat quality (sensory) parameters. Wiadomości. Zootech. R. LIII.

[33] Jeroch H., Dänicke S. (2015). Faustzahlen zur Geflügelfütterung. Geflügeljahrbuch.

[34] Wężyk S., Rosiński A., Bielińska H., Badowski J., Cywa-Benko K. (2003). A note on the meat quality of W11 and W33 White Kołuda geese strains. Anim. Sci. Pap. Rep..

[35] Liu B.Y., Wang Z.Y., Yang H.M., Wang J.M., Xu D., Zhang R., Wang Q. (2011). Influence of rearing system on growth performance, carcass traits, and meat quality of Yangzhou geese. Poult. Sci..

[36] Liu H.W., Zhou D.W. (2013). Influence of pasture intake on meat quality, lipid oxidation, and fatty acid composition of geese. J. Anim. Sci..

